# Neck-Dissection – mehr, weniger, gezielter?

**DOI:** 10.1007/s00106-025-01696-z

**Published:** 2025-12-18

**Authors:** Johannes Doescher, Johannes Zenk

**Affiliations:** Klinik für Hals‑, Nasen‑, Ohrenheilkunde, Universitätsklinikums Augsburg, Sauerbruchstraße 6, 86179 Augsburg, Deutschland

**Keywords:** Lymphknotendissektion, Wächterlymphknotenbiopsie, Immuntherapie, Bildgebung, Kopf-Hals-Tumoren, Lymph node excision, Sentinel lymph node biopsy, Immunotherapy, Diagnostic imaging, Head and neck neoplasms

## Abstract

Die Ausräumung der Halslymphknoten (Neck-Dissection) ist ein essenzieller Bestandteil der chirurgischen Therapie von Kopf-Hals-Malignomen. In den letzten Jahren konnte der für ein optimales onkologisches Ergebnis erforderliche Umfang einer Neck-Dissection zunehmend reduziert werden und teilweise auch durch alternative Verfahren wie die Wächterlymphknotenbiopsie ersetzt werden. Dies ist jedoch nur durch ein präzises Staging möglich. Hierzu werden eine verbesserte Bildgebung, molekulare Marker sowie der Einsatz von künstlicher Intelligenz angewendet. Insbesondere vor dem Hintergrund der Immuntherapie, die nun auch in der Neoadjuvanz zugelassen wurde, ist der Erhalt funktioneller lymphatischer Strukturen relevant, da sich dies positiv auf das Therapieansprechen auswirkt. Letztendlich ist die histologische Aufarbeitung der Halslymphknoten nach wie vor der Goldstandard, um die Prognose abzuschätzen und die adjuvante Therapie zu planen.

**Zusatzmaterial online:**

In der Online-Version dieses Artikels (10.1007/s00106-025-01696-z) finden Sie weiterführende Informationen und Literatur.

## Abstract (en)

Neck dissection is an essential part of surgical treatment for head and neck malignancies. In recent years, the extent of neck dissection required for optimal oncological results has been increasingly reduced and, in some cases, replaced by alternative procedures such as sentinel lymph node biopsy. However, this is only possible with precise staging. Improved imaging, molecular markers, and artificial intelligence are used for this purpose. Particularly in light of immunotherapy, which has now also been approved for neoadjuvant use, the preservation of functional lymphatic structures is relevant, as this has a positive effect on the response to treatment. Ultimately, histological examination of the cervical lymph nodes remains the gold standard for assessing prognosis and planning adjuvant therapy.

## Risikoadaptiertes Vorgehen?

Die Entfernung der zervikalen Lymphknoten (Neck-Dissection, ND) ist seit Jahrzehnten fester Bestandteil der onkologischen Therapie im Kopf-Hals-Bereich. Zunehmend wird jedoch hinterfragt, ob eine maximale Lymphknotenentfernung stets erforderlich ist oder ob ein gezielteres, risikoadaptiertes Vorgehen funktionelle und onkologische Vorteile bietet. Eine fundierte Darstellung der Grundlagen findet sich im Referat von Teymoortash und Werner, das die Basis für die aktuelle Diskussion bildet [1]. Vor diesem Hintergrund stellt sich die Frage, ob die Ausdehnung der Neck-Dissection in allen Fällen gerechtfertigt ist oder ob moderne Bildgebung, verbesserte Diagnostik und ein tieferes Verständnis der Metastasierungsmuster selektivere, weniger invasive Konzepte ermöglichen.

Dass der überwiegende Teil der Arbeiten retrospektiv angelegt ist, bringt methodische Limitationen mit sich, insbesondere hinsichtlich der Verlässlichkeit der Levelangaben der resezierten Lymphknoten: In retrospektiven Analysen sind die Zuordnung und Dokumentation der entnommenen Lymphknoten zu den jeweiligen anatomischen Regionen häufig uneinheitlich und stützen sich meist auf Operations- und Pathologieberichte, deren Präzision stark variieren kann.

## Indikationen und Ausmaß der Neck-Dissection

Die Indikationen für eine ND richten sich nach der klinischen und radiologischen Einschätzung einer zervikalen lymphonodalen Metastasierung (LNM) einerseits und dem Risiko einer okkulten Metastasierung andererseits. Die Indikation ist kaum von dem Resektionsausmaß zu trennen, da die Lage des Primarius i. d. R. das gehäufte Auftreten von Lymphknotenmetastasen in bestimmten Halsregionen bedingt. Dadurch sind nicht immer alle Halslymphknotenlevel auszuräumen, sondern je nach Lage des Primarius nur spezifische Level. Für das Risiko einer Metastasierung in ein Level wird weiterhin die von Weiss et al. im Jahr 1994 publizierte Rate von 20 % als Grenze für die Indikation zur ND angesehen und von den Autoren der meisten Studien zu diesem Thema als Benchmark verwendet [2].

**Abb. 1:**
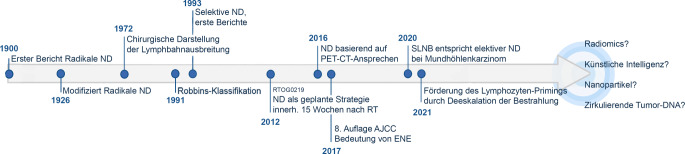
Überblick über die Meilensteine in der Entwicklung der Neck-Dissection. *AJCC* American Joint Committee on Cancer, *ENE* extranodale Ausbreitung („extra nodal extension“), *ND* Neck-Dissection, *PET-CT* Positronenemissionstomographie-Computertomographie, *RT* Radiotherapie; *SLN* Wächterlymphknotenbiopsie („sentinel lymph node biopsy“). (Graphikdesign durch I. Gutekunst)

Für Patienten mit einer klinisch eindeutigen zervikalen Metastasierung wird die Entfernung der metastatisch involvierten Level empfohlen. Das darüber hinausgehende Ausmaß der ND ist seit Langem Gegenstand der Diskussion und Anlass für zahlreiche Studien. Die Empfehlung zur bedingungslosen Indikation für eine radikale ND wurde schon seit geraumer Zeit verlassen und durch das Konzept der modifiziert radikalen ND (MRND) ersetzt (Abb. 1). Aber auch die MRND wird meist nur noch in Fällen einer ausgeprägten Metastasierung mit kapselüberschreitendem Wachstum (extranodale Ausbreitung, ENE+, cN3b) angewandt, und es wird häufig die gezielte Ausräumung betroffener Level unter Einbezug von Leveln mit einem hohen Risiko für eine okkulte Metastasierung empfohlen (selektive ND, SND) [1]. Auch wenn der Eingriff insgesamt eine geringe Morbidität aufweist, wird kontinuierlich an einer Verfeinerung der chirurgischen Techniken gearbeitet [3]. Auch eine Reduktion des Ausmaßes der ND hat positive Auswirkungen u. a. auf die Morbidität des Eingriffs. So lassen sich Komplikationen wie Schulterdysfunktion, Schulterschmerzen, Lymphödeme, Chylusfisteln und, seltener, Nervenparesen und Thrombosen der V. jugularis interna vermeiden [4].

### Mundhöhlenkarzinome

Die ND bei Mundhöhlenkarzinomen ist im Vergleich zu anderen Lokalisationen im Kopf-Hals-Bereich ein viel beachtetes und beschriebenes Thema. Das mag an der Überschneidung zweier Fachbereiche, der Mund-Kiefer-Gesichts-Chirurgie und der Hals-Nasen-Ohren-Heilkunde, liegen. Auch die entsprechende deutsche Leitlinie ist, neben Beteiligung anderer Fachgebiete, von Vertretern beider Fachgesellschaften verfasst worden. In der aktuell gültigen Leitlinie von 2021 wird klar formuliert, dass sowohl bei cN0- als auch bei cN+-Status prinzipiell eine ND indiziert ist. Allerdings sind die Aussagen zum Ausmaß der ND weit weniger eindeutig und lassen teilweise einen großen Interpretationsspielraum zu. Dies zeigt, wie wenig Evidenz die bisherige Studienlage generieren konnte. Der Bewertung des Expertengremiums der Leitlinie nach kann eine MRND anstelle einer RND nur in ausgewählten Fällen empfohlen werden. Dem gegenüber kommen die Autoren zu dem Schluss, dass das Ausmaß der ND in Kombination mit einer adjuvanten Radio- bzw. Radiochemotherapie reduziert werden kann. Beim Vorliegen eines einzelnen metastatischen Lymphknotens wurde im Expertenkonsens die Möglichkeit zu einer SND eröffnet, solange die ND mindestens ein Level weiter nach kaudal geführt wird, als die Metastase lokalisiert ist. Für Fälle mit unauffälligem Lymphknotenstatus (cN0) ist eine SND der Level I–III ausreichend. Ob bilateral oder nur ipsilateral operiert werden muss und ob Level IIb miteingeschlossen werden sollte, richtet sich laut der Leitlinie nach der Lokalisation des Primarius [5].

In einer großen Studie anhand von Daten aus der National Cancer Database (NCDB) wurden 25.846 Patienten mit Mundhöhlenkarzinomen hinsichtlich der lymphogenen Metastasierung analysiert. Zum Zeitpunkt der Erstdiagnose lag in 26,7 % der Fälle eine klinische LNM vor, wobei das Risiko mit höheren T‑Stadien zunahm. Die Mundhöhle wurde von den Autoren in 8 verschiedene Unterbezirke unterteilt. Am häufigsten betroffen waren Fälle mit Primarien des retromolaren Trigonums, gefolgt von Karzinomen des Mundbodens. Es lag in 19,6 % eine falsch-positive Einschätzung klinischer LNM vor. Umgekehrt konnte eine okkulte Metastasierungsrate von insgesamt 25,1 % der cN0-Fälle festgestellt werden, wobei T1-Karzinome in 16,6 % der Fälle betroffen waren. Auch hier waren wieder Karzinome des retromolaren Bezirks führend (28,8 %), gefolgt von Karzinomen der Zunge (27,5 %) [6]. Die Studie zeigt eine relative Bandbreite sowohl der klinisch apparenten LNM als auch der okkulten Metastasierungsrate abhängig von den jeweiligen Unterbezirken des Primarius. Zudem wird klar, dass die präoperative Einschätzung der zervikalen Lymphknoten nicht immer ganz einfach ist und falsch-positive Ergebnisse dann zu einer möglichen Übertherapie führen. Neben diesen Parametern, welche ein generelles Risiko für eine LNM abschätzen können, stellt sich die Frage, welche Level am ehesten davon betroffen sind. Hierfür bedarf es deutlich präziserer Daten. Eine Metaanalyse über 2323 Patienten aus 17 Studien konnte bestätigen, dass Level II mit 20 % am häufigsten betroffen ist. Dabei lag eine geringe Heterogenität zwischen den analysierten Studien vor. Ebenfalls mit niedriger Heterogenität wurde eine LNM-Frequenz von 12 % in Level I und 10 % in Level III berechnet. In Level IV und Level V wurden nur selten LNM detektiert (2 % bzw. 1 %) [7]. Dies unterstützt weiter das gängige Verständnis, dass Level I–III ein besonderes Risiko für LNM zugeschrieben wird. Allerdings wurde keine weitere Unterteilung in Level Ia und Ib bzw. Level IIa und IIb vorgenommen. Zudem wurde keine Stratifizierung des Risikos anhand der Lokalisation und der Größe des Primarius vorgenommen. Eine detaillierte Analyse führten Kollegen aus der Schweiz an Patientendaten aus 2 Zentren durch. Die Inkorporation multiparametrischer Daten, inkl. der Seite der LNM und der Lage des Primarius in Bezug zur Mittellinie, führt zu einer komplexen Auswertung. Um diese auch für die eigenen Patienten verständlicher zu machen, veröffentlichten die Autoren eine Internetplattform, auf welcher die Daten öffentlich zugänglich gemacht wurden (https://lyprox.org). Hier besteht auch die Möglichkeit, das Risiko für individuelle Patienten anhand der publizierten Daten abzuschätzen. Primarien der Zunge metastasierten am häufigsten in Level II (35 %) und weniger häufig in Level I und II (15 und 16 %). Tumoren des Zahnfleischs und der Wangenschleimhaut hatten gehäuft Metastasen in Level I (31 %), wohingegen Mundbodenkarzinome gleichermaßen in Level I und II metastasierten. Risikofaktoren für eine kontralaterale Metastasierung waren die Größe des Primarius, seine Nähe zur Mittellinie und das Vorliegen ipsilateraler LNM [8]. Für diese Konstellation lässt sich eine Empfehlung zur kontralateralen ND beim Vorliegen ipsilateraler Metastasen ableiten [9].

Einig sind sich die meisten Studien darin, dass die ND bei Mundhöhlenkarzinomen Level I mit umfassen sollte. Ob hierbei die Gl. submandibularis (GSM) entfernt werden sollte, wird kontrovers betrachtet. Ein Erhalt der Drüse ist funktionell nicht zu unterschätzen, da beide GSM für 70 % des Speichelvolumens verantwortlich sind [1]. Neuere Studien konnten eine direkte Infiltration der GSM in 0,7–1,32 % zeigen, wobei in der Hälfte der Fälle der Primarius die Drüse direkt infiltrierte [10–12]. Die Empfehlung der Autoren lautete jeweils, dass es onkologisch sicher und funktionell vorteilhaft sei, die Drüse zu schonen. Als Gegenargument hierzu wird der eingeschränkte Zugang zu den perimandibulären Lymphknoten angeführt [1]. Wobei dies technisch an sich kein Problem darstellt, da die Drüse an ihrer Kapsel gut aus der Umgebung gelöst werden kann und so auch verdeckte Lymphknotengruppen zugänglich werden.

Auch bei den aktuellen Studien zeigt also sich eine große Unsicherheit bzgl. des Ausmaßes einer ND für cN+-Mundhöhlenkarzinome. Dieser Unsicherheit muss zum einen durch eine zunehmende Präzision im klinischen Staging und zum anderen durch prospektive randomisierte Studien begegnet werden.

Die Rate sog. okkulter Lymphknotenmetastasen bei Mundhöhlenkarzinomen ist weiterhin Gegenstand zahlreicher Studien. Es zeigt sich hier eine große Bandbreite von 7–40 %, was u. a. an der Heterogenität der eingeschlossenen Tumoren hinsichtlich Größe und Lokalisation liegt [6, 13–16]. Einen Überblick über die neueren Erhebungen bietet Tab. 1. Ob und inwieweit eine ND bei cN0 sinnvoll ist, hängt letztlich von der prognostischen Bedeutung möglicher Lymphknotenmetastasen ab. Gegenstand dieser Analysen sind v. a. kleinere Karzinome, da hier die Wahrscheinlichkeit für eine LNM geringer und die Unsicherheit hinsichtlich einer elektiven ND (END) größer ist. Für 3886 Patienten mit cT1-Karzinomen lag die zervikale Kontrollrate innerhalb von 5 Jahren bei 96 % nach END und 90 % nach einer Watch-and-Wait-Phase. Das krankheitsspezifische Überleben war zwar nur um 1 % schlechter (93 vs. 92 %), dies war jedoch aufgrund der großen Fallzahl statistisch signifikant, was auch von anderen Analysen unterstützt wird [17, 18]. Noch deutlicher wird dieser Unterschied bei Patienten mit cT2-Tumoren. Hier wurde ein signifikant längeres 5‑Jahres-Überleben von 73,6 % nach END gegenüber 64,5 % nach Beobachtung errechnet. Das verbesserte Überleben war auch in einer multivariaten Analyse signifikant mit der Durchführung einer END assoziiert [19]. Die Autoren leiten aufgrund der enormen Fallzahl eine Indikation für eine END für cT2-Mundhöhlenkarzinome ab.

**Tab. 1:** Auswahl von Studien zur Rate okkulter Lymphknotenmetastasen beim Mundhöhlenkarzinom.

Referenz	Jahr	Gesamtzahl cN0 und END	Anzahl okkulter Lymphknotenmetastasen (%)
Knopf A et al. [39]	2020	142	57 (40,1)
Haidari S et al. [13]	2022	226	16 (7,1)
Carey R et al. [6]	2023	11.334	2849 (25,1)
Mrosk F et al. [16]	2024	139	27 (19,4)
Duvernay J et al. [15]	2025	389	98 (25,2)

*END* elektive Neck-Dissection

Neben der Größe spielt in der Literatur zunehmend auch die Invasionstiefe („depth of invasion“, DOI) des Primarius eine Rolle. Diese ist zwar in der aktuell gültigen TNM-Klassifikation teilweise integriert worden, jedoch gibt es darüber hinausgehende Betrachtungen insbesondere hinsichtlich der Risikoabschätzung von okkulten LNM. Entsprechend des AJCC-Staging-Systems von 2017 sind Tumoren bis zu 5 mm DOI als T1, zwischen 6–10 mm als T2 und darüber als T3 zu klassifizieren [20]. Die Empfehlungen bezüglich einer durch die DOI gestützten Indikation zur END gehen auch in den Leitlinien auseinander. So nennt die deutsche Leitlinie 3 mm, die amerikanische beispielsweise 4 mm als Cut-off-Wert für eine END in einem bestimmten Kontext. Eine Studie an 300 Patienten mit cT1/cT2-cN0-Mundhöhlenkarzinomen überprüfte den Cut-off-Wert von einer DOI ≥ 4 mm. Es wurde eine signifikante Assoziation eines DOI ≥ 4 mm mit dem Vorliegen einer okkulten LNM festgestellt. Dies wurde in einer multivariaten Analyse als unabhängig bestätigt. In einer Receiver-Operating-Characteristic(ROC)-Analyse für verschiedene Unterbezirke wurde ein optimaler Cut-off-Wert der DOI von ≥ 5 mm für Zungenkarzinome, ≥ 4 mm für Mundbodenkarzinome und ≥ 7 mm für Wangenschleimhautkarzinome ermittelt [21]. Andere Studien bestätigen die Bedeutung der DOI. Der optimale Cut-off-Wert differiert jedoch von Studie zu Studie und liegt je nach Unterbezirk zwischen ≥ 4 und ≥ 7 mm [16, 22–24].

**Abb. 2:**
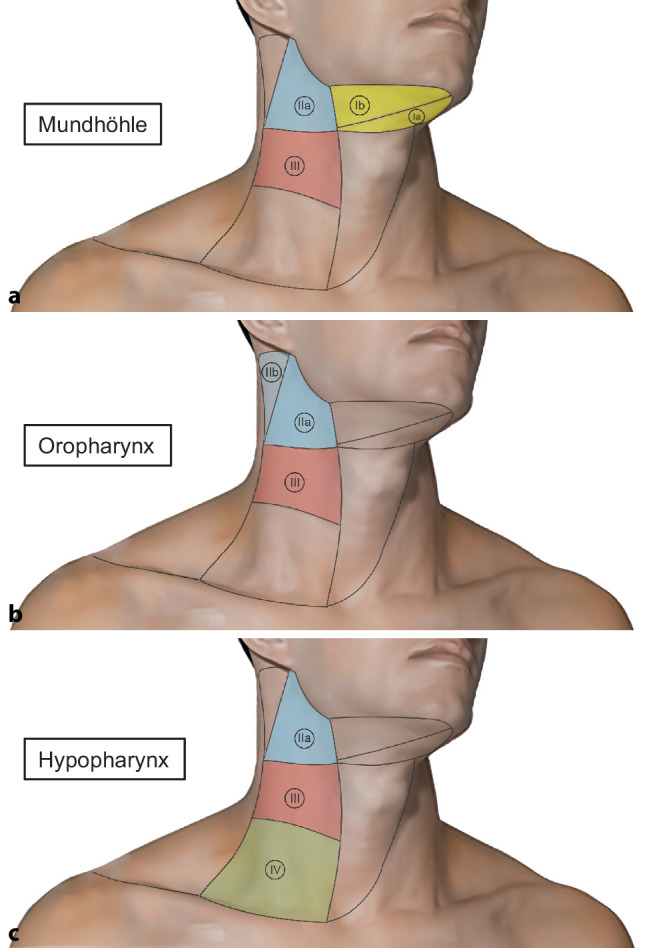
Mindestumfang einer elektiven Neck-Dissection (END) entsprechend der aktuellen Literatur. **a** Bei Mundhöhlenkarzinomen mindestens Level I, IIa und III, **b** bei Oropharynxkarzinomen mindestens Level IIa und III, Level IIb erst ab T3, **c** bei Hypopharynxkarzinomen mindestens Level IIa, III und IV. (Graphikdesign durch I. Gutekunst)

Das Ausmaß der END für cN0-Karzinome sollte entsprechend den bisherigen Ausführungen mindestens die Level I–III beinhalten (Abb. 2a). Die Ausdehnung der END auf Level IV und V wird weiterhin diskutiert, wobei sich zunehmend die Empfehlung zu einer Schonung dieser Level durchsetzt. Eine große Metaanalyse konnte bei 11 Studien und 498 Patienten in nur 2,8 % der Fälle eine okkulte LNM in Level IV feststellen [25]. Auch hinsichtlich des Überlebens konnte in einer Studie speziell zu Zungenkarzinomen gezeigt werden, dass für cT1/cT2-cN0-Patienten mit einer umschriebenen ND der Level I–III kein statistischer Unterschied erzielt werden konnte verglichen mit Patienten, bei denen aufgrund eines cN+-Status eine MRND erfolgt war [26]. Um die Morbidität weiter zu senken und die END noch präziser zu gestalten, wird weiter die Relevanz von Level IIb hinterfragt. Insgesamt ist die LNM-Rate in diesem Unterbezirk gering und reicht von 0–5,6 %, wobei über Metastasen in Level IIb ausschließlich im Zusammenhang mit einer Metastasierung in Level IIa berichtet wurde [15, 27]. Wenn man das Datenset von Ludwig et al. exploriert, findet sich eine Metastasierungsrate von 4 % in Kombination mit Level IIa und 1 % ohne LNM in Level IIa [8]. Bislang gibt es keine eindeutigen Daten bzgl. der Lokalisation des Primarius und eines spezifischen Risikos einer LNM in Level IIb, weswegen die Empfehlung der deutschen Leitlinie einer Mitausräumung von Level IIb bei Zungenkarzinomen zu hinterfragen ist.

Die Entscheidung, ob eine bilaterale END oder eine ipsilaterale END durchgeführt werden muss, sollte anhand der Lokalisation des Primarius getroffen werden. In einer Studie, in welcher insbesondere bilateral und ausschließlich kontralateral auftretende LNM betrachtet wurden, konnte gezeigt werden, dass dieses Risiko v. a. signifikant für Tumoren nahe der Mittellinie, nahe dem Mundboden und dem Oberkiefer sowie für große T4a-Karzinome besteht. Wie auch durch Ludwig et al. gezeigt, treten kontralaterale LNM gehäuft beim Vorliegen ipsilateraler Metastasen auf [8, 9].

### Oropharynxkarzinome

Für die Diagnostik und Therapie von Oropharynxkarzinomen ist erst kürzlich eine Leitlinie für den deutschsprachigen Raum erschienen. Eine elektive ND (END) bei cN0-Patienten sollte demnach die Level II–IV umfassen. Hierbei ist eine Schonung des N. accessorius zur Verbesserung der Lebensqualität explizit gefordert. Bei kleinen (cT1/cT2-)Tumoren, welche streng lateral der Mittellinie wachsen, wird eine ipsilaterale ND empfohlen, bilateral und unabhängig von der Größe sollte operiert werden bei mittelliniennahen sowie Weichgaumen- und Zungengrundkarzinomen. Der p16-Status spielt für die Empfehlung zur Durchführung und Umfang einer END bei cN0-Status aktuell keine Rolle. Die Wächterlymphknotenbiopsie kann zum jetzigen Zeitpunkt aus Sicht des Expertengremiums nicht empfohlen werden. Hinsichtlich der kurativen ND bei cN+-Tumoren lässt die Leitlinie relativ viel Spielraum. Analog zur END bei cN0-Tumoren wird mindestens eine Ausräumung von Level II–IV gefordert. Ob ipsi- oder bilateral operiert werden sollte, richtet sich ebenfalls nach der Lage des Primarius und weniger nach dem Vorliegen einer ipsilateralen LNM [28].

Um die Frage nach einer möglichen Vermeidung bzw. Reduktion des Ausmaßes einer END zu beantworten, müssen die Raten okkulter Metastasen betrachtet werden. Hier zeichnet sich analog zur Mundhöhle ab, dass die Bewertung des Risikos zunehmend detaillierter wird und einzelne Unterbezirke des Oropharynx bzw. biologische Unterschiede gesondert betrachtet werden. Auf humanes Papillomavirus positive (HPV+-)Karzinome sind bekannt für eine frühe und ausgeprägte LNM bei gleichzeitig guter Prognose, was sich im aktuell gültigen AJCC-Staging-System widerspiegelt [20]. In einer Auswertung der SEER-Datenbank (Surveillance, Epidemiology, and End Results Program) mit Fokus auf kleine, primär chirurgisch therapierte Oropharynxkarzinome (cT1–2), wurden 470 HPV+ mit 375 HPV-Fällen verglichen. Nach „propensity score matching“ wurde eine Überlebensanalyse durchgeführt und innerhalb der Subgruppen nach durchgeführter END und klinischer Beobachtung stratifiziert. Hier zeigte sich im 3‑Jahres-Überleben ein signifikanter Überlebensvorteil für HPV+-Patienten, welche eine END erhalten hatten (89,7 vs. 78,7 %). Für HPV-Patienten war eine END weder ein unabhängiger prognostischer Faktor noch assoziiert mit einem verbesserten Überleben [29]. Okkulte Lymphknotenmetastasen bei HPV+-Karzinomen treten v. a. im Level II (27 %), gefolgt von Level III (8,7 %) und Level IV (4 %) sowie 3,3 % in Level I auf. Ein signifikanter Zusammenhang mit T‑Stadien oder Lokalisation des Primarius konnte nicht gefunden werden. Die Autoren der Studie schlagen auf Basis ihrer Daten eine Reduktion der END für HPV+-Tumoren auf Level II–III vor, da das Risiko einer okkulten LNM in Level I und IV relativ niedrig zu sein scheint (Abb. 2b; [30]). Auch bei HPV-Karzinomen liegt die Rate für okkulte Lymphknotenmetastasen laut einer neueren Studie bei 4,8–8,1 % in Level I und nur 3,2 % in Level IV. Die Integration von Level IV in die END führte zudem zu keinem Überlebensvorteil. Somit könnte auch bei HPV-Tumoren Level IV geschont werden [31].

Weiter stellt sich die Frage, ob nun Level IIb mitadressiert werden muss oder geschont werden kann. Eine doppelblinde randomisierte Studie konnte klar herausarbeiten, dass die Ausräumung von Level IIb mit einer deutlichen Einschränkung der Schulterbeweglichkeit, der Nervenleitgeschwindigkeit des N. accessorius und letztlich einem signifikant niedrigeren Neck-Dissection-Impairment-Index einhergeht [32]. Daher sollte, wenn onkologisch möglich, Level IIb geschont werden. Die Varianz hinsichtlich einer okkulten Metastasierung in Level IIb ist groß und reicht von 2,5–20 % für cN0-Fälle und 5–50 % für cN+-Fälle. Eine detaillierte Multizenterstudie fand in 3,3 % der cN0-Fälle eine okkulte LNM in Level IIb, wovon nur in einem Fall eine isolierte Metastase in diesem Sublevel auftrat. Das Risiko für eine Beteiligung von Level IIb stieg auf 13,8 % bei cN+-Fällen an [33]. Die Datenlage ist insgesamt noch so heterogen, dass eine Schonung von Level IIb aktuell nur für cT1/cT2-cN0-Tumoren erfolgen sollte, was sich auch mit den Empfehlungen der deutschen Leitlinie deckt (Abb. 2b).

Vergleichsweise häufig diskutiert wird die Frage, ob eine ipsilaterale oder bilaterale END bei cN0-Oropharynxkarzinomen durchgeführt werden sollte. Eine Übersicht über die Ergebnisse verschiedener Studien findet sich in Tabelle S1. Nicht alle Studien liefern hier gleichermaßen detaillierte Daten und sind teilweise sehr fokussiert auf bestimmte Aspekte des Oropharynxkarzinoms. Die Rate okkulter kontralateraler Metastasen reicht von 0,6–11,1 % [34–39]. Hierbei weisen nur bis zu 1,7 % der Fälle mit einem ipsilateralen cN0-Status eine kontralaterale LNM auf [4, 34–36, 38]. Bei Vorliegen von positiven Lymphknoten ipsilateral steigt die Rate auf bis zu 13,04 % [4]. Andere Autoren sehen keinen Zusammenhang zwischen der Lokalisation des Primarius und dem vermehrten Auftreten kontralateraler LNM, wobei die meisten Studien ausschließlich Tumoren mit streng lateralem Wachstum und einige Studien auch ausschließlich Tonsillenkarzinome betrachtet hatten. Eine Stratifizierung nach HPV-Status wurde in keiner Studie vorgenommen. Die Übersicht in Tabelle S1 macht deutlich, dass dies auch kein entscheidender Faktor zu sein scheint. Tendenziell kommt es bei höheren T‑Stadien eher zu einer kontralateralen LNM, jedoch sind die Ergebnisse hierzu nicht eindeutig [35–37, 39].

Eine weitere Studie über 71 Patienten mit mehrheitlich HPV+-Tonsillenkarzinomen unterschiedlicher Stadien, welche alle streng ipsilateral therapiert wurden, wertete die Daten hinsichtlich regionaler Kontrolle insbesondere der nicht therapierten Halsseite aus. Die Therapiemodalitäten reichten von primärer Radiatio über alleinige Chirurgie bis hin zu einer Kombination aus Chirurgie und Radio(chemo)therapie. Im Follow-up kam es selten zu ipsilateralen Lymphknotenrezidiven (8 %) und nie zu kontralateralen Rezidiven [40, 41]. Diese Ergebnisse unterstützen ein streng ipsilaterales Vorgehen zumindest bei Tonsillenkarzinomen, was auch die adjuvante Therapie miteinschließen sollte. Allerdings scheinen auch andere Lokalisation nur ein sehr überschaubares Risiko für eine kontralaterale Lymphknotenmetastasierung bei ipsilateralem cN0-Status aufzuweisen.

Weiterhin wird in der aktuellen deutschsprachigen Leitlinie unter bestimmten Umständen auch für cN+-Karzinome die Möglichkeit einer einseitigen SND eingeräumt. Dieses Vorgehen kann nach der bisherigen Datenlage für lateral wachsende Tumoren mit einzelnen ipsilateralen Lymphknoten verfolgt werden. Eine Nähe des Primarius zur Mittellinie und insbesondere größere oder mehrere ipsilaterale Lymphknotenmetastasen weisen allerdings ein erhöhtes Risiko für eine kontralaterale Metastasierung auf (25–100 %) und sollten daher bilateral therapiert werden [38].

### Hypopharynxkarzinome

Die gemeinsame Leitlinie für Oro- und Hypopharynxkarzinome empfiehlt auch für Letztere eine END bereits für kleine T‑Stadien. Es wird eine Ausräumung mindestens der Level IIa, III und IV gefordert (Abb. 2c). Bilateral sollte operiert werden, wenn die Tumoren mittelliniennah gelegen sind, eine tiefe Tumorinfiltration aufweisen oder ≥ T2 sind. Insgesamt wird bei Hypopharynxkarzinomen in der Mehrzahl der Fälle jedoch bereits bei Erstdiagnose eine LNM diagnostiziert. Das Ausmaß der therapeutischen ND richtet sich analog zu den Oropharynxkarzinomen nach der Lage des Primarius (lateral vs. mittelliniennah) und dem Ausmaß des Lymphknotenbefalls. Bei fortgeschrittenen Karzinomen wird zudem die Möglichkeit einer Ausräumung prätrachealer sowie paratrachealer Lymphknoten und ggf. eine Resektion der Schilddrüse genannt [28].

Verglichen mit anderen Lokalisationen, wurden in den letzten Jahren nur sehr wenige Arbeiten zur Rolle der ND bei Hypopharynxkarzinome publiziert. Ein möglicher Grund hierfür ist, dass häufig ein organerhaltendes Konzept mit einer primären Radiochemotherapie für die Behandlung dieser Tumoren gewählt wird.

Eine Studie aus Japan wertete die Daten von 221 Patienten mit mehrheitlich cT1/cT2-Tumoren aus. 10 % der cT1-, 30 % der cT2- und 50 % der cT3-Tumoren wiesen eine LNM auf. Dabei wurde eine END nur bei 4,1 % der cN0-Fälle durchgeführt. Trotzdem traten in der Gruppe ohne ND keine gehäuften Lymphknotenrezidive auf. Eine postoperative Radio(chemo)therapie führte nur bei pN3b-Fällen zu einer signifikanten Verbesserung der regionalen Kontrolle, jedoch ohne Einfluss auf das Gesamtüberleben. Die Autoren schlussfolgern, dass für kleine cN0-Hypopharynxkarzinome möglicherweise keine ND notwendig ist [42]. Die Studie bleibt allerdings die Antwort nach der Rate okkulter LNM schuldig. Hier fanden Deuß et al. eine Rate von 41,7 % bei Fällen mit cN0-Status und eine Gesamtrate von 72,2 % aller Fälle. Die Rate okkulter kontralateraler LNM war ebenfalls hoch und lag für T1/2 bei 22,2 % und für T3/4 bei 27,3 %. Die Autoren geben zudem eine Übersicht über die Literatur, welche die beobachteten LNM-Raten weitgehend bestätigt, wenn auch in etwas geringerer Ausprägung [43].

Da Hypopharynxkarzinome zu einem relevanten Anteil nicht primär chirurgisch therapiert werden, stellt sich die Frage nach dem Umgang mit großen Lymphknotenmetastasen (klinisch ENE+), die ggf. nicht vollständig auf eine Radiatio ansprechen. Hier besteht die Option einer Upfront-Neck-Dissection (UND). Es konnte gezeigt werden, dass die Durchführung einer UND das Risiko für ein lokoregionäres Rezidiv signifikant senkt (Hazard Ratio, HR: 0,382) sowie das Gesamtüberleben verbessert (HR: 0,436) [44]. Dies scheint insbesondere bei Patienten mit einem bereits klinisch und radiologisch vermuteten extrakapsulären Wachstum relevant zu sein [45, 46]. In einer durch „propensity score matching“ balancierten Studie wurden eine ausgewogene Anzahl Patienten mit und ohne ENE hinsichtlich des Outcomes einer UND verglichen. Die radiologische Vorhersage einer ENE war relativ präzise für Lymphknoten ≥ 2 cm (89,9 %) und weniger verlässlich für Lymphknoten < 2 cm (67,5 %). Eine isolierte Betrachtung der ENE+-Fälle ergab einen signifikanten Einfluss einer UND auf das krankheitsfreie 5‑Jahres-Überleben (43,9 vs. 16 %), was sich ebenfalls auf das Gesamtüberleben auswirkte und von der regionalen Rezidivrate abhängig war [47].

### Larynxkarzinome

Die Indikation zur ND sowie das Ausmaß einer ND bei Larynxkarzinomen richtet sich neben dem T‑Stadium v. a. auch nach der Untereinheit des Larynx, in welcher der Primarius lokalisiert ist. Laut der aktuell gültigen deutschen Leitlinie kann bei kleinen T1-Tumoren der Glottis und der Supraglottis auf eine END bei cN0-Status verzichtet werden. Bei einem T2-Tumor der Glottis ist eine END nicht zwangsläufig indiziert, kann aber erwogen werden. Beim supraglottischen Karzinom sollte ab T2 eine END erfolgen. Ob bilateral oder ipsilateral disseziert werden sollte, hängt von der Lage des Tumors zur Mittellinie ab. Eine END sollte sich von Level IIa bis IV erstrecken. Die therapeutische ND sollte sich nach der Ausprägung der zervikalen Metastasierung richten. Hier wird die Möglichkeit von einer selektiven ND bis hin zu einer radikalen ND eingeräumt. Falls präoperativ bereits eine hohe Wahrscheinlichkeit zur adjuvanten R(C)T besteht, sollte bei Vorliegen eines cN0-Status der kontralateralen Seite diese nicht disseziert werden [48].

**Abb. 3:**
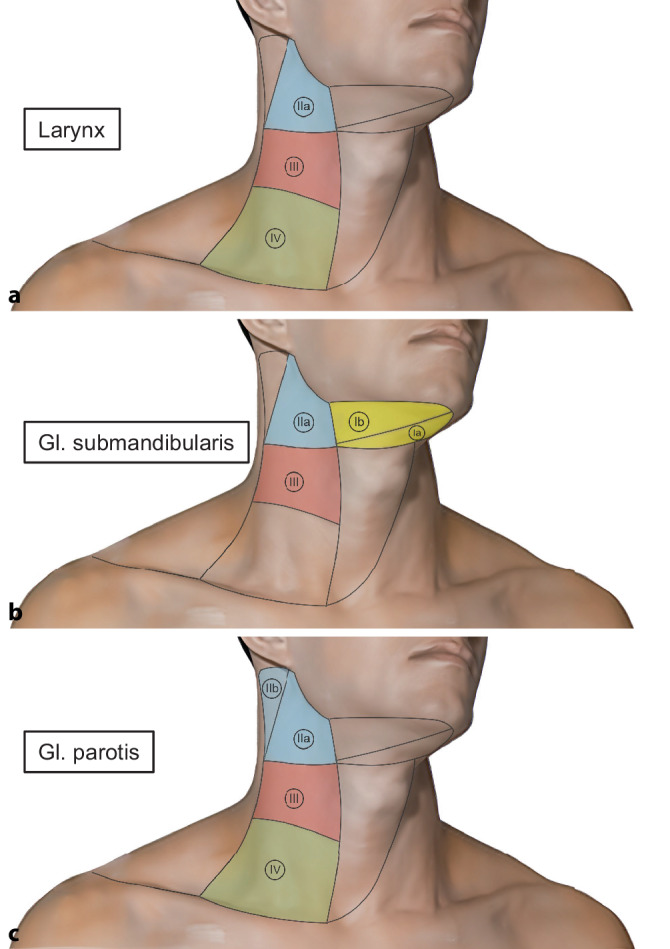
Mindestumfang einer elektiven Neck-Dissection (END) entsprechend der aktuellen Literatur. (**a** Bei Larynxkarzinomen mindestens Level IIa und III, Level IV erst ab T3, **b** bei Karzinomen der Gl. submandibularis mindestens Level I, IIa und III; **c** bei Parotiskarzinomen mindestens Level IIa–IV). (Graphikdesign durch I. Gutekunst)

In einer umfassenden Metaanalyse von 36 Studien aus dem Jahr 2020 wurde eine Rate okkulter Lymphknotenmetastasen von 18,7 % erfasst. Bei supraglottischen Karzinomen lag die Inzidenz über alle T‑Stadien für eine okkulte LNM bei 19,9 % und bei 18,4 % für T1/T2-Tumoren. Bei glottischen T1/T2-Karzinomen lag die Rate bei 8 %. Weiter spezifiziert konnte für T1-Tumoren eine Gesamtrate von 4,8 %, für glottische T2-Karzinome von 4,7 % und für supraglottische T2-Karzinome von 16,5 % ermittelt werden. Bei Tumoren ab T3 ist die strenge Trennung nach supraglottischen und glottischen Karzinomen meist nicht mehr möglich. Die Rate einer okkulten LNM wurde in der Metaanalyse mit 23,4 % berechnet. Insgesamt stützen die Daten die aktuell gültigen Empfehlungen. Lediglich der Verzicht auf eine END bei supraglottischen T1-Larynxkarzinomen wird von den Autoren aufgrund mangelnder Daten als fraglich eingestuft [49]. Neuere Arbeiten fanden bei supraglottischen T1-Karzinomen relativ hohe Raten einer okkulten LNM (14–16,5 %) und empfehlen eine END [50, 51]. Im Gegensatz dazu berichten Zorzi et al. von einer geringen Lymphknotenrezidivrate für supraglottische Larynxkarzinome im Stadium T1–T3 nach Resektion ohne END [52]. Eine Analyse von glottischen Larynxkarzinomen im Stadium T1–2, welche in der NCDB erfasst wurden, konnte einen Vorteil einer END für High-Grade-Karzinome herausarbeiten. Zwar führte eine END generell zu keinem Überlebensvorteil, aber das Vorliegen von okkulten Lymphknoten zu einem signifikanten Überlebensnachteil. Unabhängig prädiktiv für eine okkulte LNM war das Vorliegen einer High-Grade-Pathologie. Die Autoren folgern aus ihren Daten, dass für wenig bzw. undifferenzierte glottische Karzinome im Stadium T1–2 eine END erwogen werden sollte [53]. Für Karzinome der Subglottis ist die Datenlage deutlich dünner, da diese häufig primär strahlentherapeutisch behandelt werden. Aus der NCDB findet sich eine Gesamtrate von 26,5 % mit aufsteigendem Risiko abhängig vom T‑Stadium [54]. Bezüglich der Ausdehnung der ND sind die Raten in Level IIb (0,5 %) und Level IV (0,9–2 %) so niedrig, dass eine Ausdehnung der END auf Level IIb generell nicht und auf Level IV nur ab T3 empfohlen wird (Abb. 3a; [49, 55]).

Kontroversen bestehen bei der Ausdehnung einer END im Fall einer Laryngektomie. Bei der Präparation des Kehlkopfs bietet sich eine bilaterale SND der Level II–IV technisch gesehen an. Allerdings besteht weitgehend Einigkeit darüber, dass auch hier Level IIb nicht mitausgeräumt werden sollte [49, 56]. Hinsichtlich einer END der kontralateralen Seite gibt es weniger klare Daten. Eine Einteilung der Tumorausdehnung in 4 Kategorien ergab ein relevantes Risiko für eine kontralaterale okkulte LNM bei T3/T4-Karzinomen erst ab einem klaren Überschreiten, nicht aber für ein bloßes Erreichen der Mittellinie (0 vs. 18,8 %) [56]. Insgesamt scheint die Rate kontralateraler LNM allerdings stark zu schwanken (3,8–21,6 %) [56, 57]. Eine Analyse aus Japan konnte in einer Kohorte von 1218 Patienten keinen Überlebensvorteil einer bilateralen gegenüber einer ipsilateralen END finden. Auch ein vollständiger Verzicht auf eine ND war hinsichtlich des Gesamtüberlebens und des rezidivfreien Überlebens einer END nicht unterlegen. Allerdings waren über 40 % dieser Patienten über 80 Jahre alt, was generell mit einer schlechteren Prognose assoziiert ist und daher das Ergebnis beeinflussen könnte [58]. Insgesamt kann ein Verzicht auf eine END der Gegenseite bis dato nicht empfohlen werden bzw. nur analog zur deutschen Leitlinie in Erwartung einer adjuvanten R(C)T erfolgen.

### Speicheldrüsenkarzinome

Speicheldrüsenkarzinome stellen aufgrund ihrer Seltenheit und der Heterogenität der histologischen Entitäten, verbunden mit teils gravierenden Unterschieden im klinischen Verhalten, eine große Herausforderung bei der Etablierung von Behandlungsalgorithmen auch der Halslymphknoten dar. Das französische Netzwerk für seltene Kopf-Hals-Tumoren (REFCOR) hat im Jahr 2024 eine Konsensusleitlinie speziell zu diesem Thema verfasst. Für Tumoren mit einer klinisch apparenten Lymphknotenmetastasierung wird eine ipsilaterale ND empfohlen. Sowohl für Tumoren der Gl. parotis als auch der Gl. submandibularis sollte die ND Level I–V umfassen. Der Umfang bei Tumoren kleiner Speicheldrüsen sollte sich nach der Primärlokalisation richten und an den Empfehlungen für diese orientieren. Für cN0-Fälle wird darauf hingewiesen, dass die Literatur heterogen ist. Das Expertengremium schlussfolgert eine generelle Empfehlung zur END. Diese kann laut Autorenmeinung nur dann durch klinische Kontrollen ersetzt werden, wenn es sich um Low-Grade-T1/T2-Tumoren bzw. adenoidzystische Karzinome (ACC) im Stadium T1–2 ohne Infiltration der Mukosa handelt. Davon ausgenommen sind sekretorische Karzinome und Primarien der Gl. submandibularis. Bei Letzteren sollte zumindest eine SND von Level Ib erfolgen [59]. Die aktuelle deutsche Leitlinie empfiehlt in der cN+-Situation ebenfalls eine MRND ohne Auslassen spezifischer Level. Hinsichtlich der END ist die Leitlinie noch zurückhaltender formuliert. Abweichend von der französischen Leitlinie sollte nicht bei Karzinomen der Gl. submandibularis, sondern der Gl. parotis eine END erfolgen. Ebenfalls abweichend wird herausgestellt, dass bei lokal fortgeschrittenen ACC eine END unabhängig von der Lokalisation durchgeführt werden sollte. Eine Empfehlung für andere Entitäten wird nicht formuliert [60].

Die Raten okkulter LNM schwanken stark und reichen von 2 % bis über 50 % je nach Histologie, Grading und Größe [61, 62]. Dies variiert zudem je nach Lokalisation des Tumors. Für Tumoren der Gl. parotis finden sich Raten zwischen 15–40 %, im Bereich der Gl. submandibularis weisen 20–35 % Tumoren eine okkulte LNM auf. Auch die Verteilung innerhalb der Level variiert entsprechend. So findet sich bei Parotiskarzinomen eine okkulte LNM in Level I–V mit Raten von bis zu 23 % in Level IV und bis zu 18 % in Level V, wohingegen Karzinome der Gl. submandibularis vornehmend in Level I–III metastasieren. Insbesondere bei Letzteren scheint die Histologie weniger eine Rolle für das Risiko einer okkulten LNM zu spielen, da hier alle Entitäten gleichermaßen häufig Metastasen aufweisen [63]. Eine große, systematische Metaanalyse analysierte das Auftreten von okkulten Lymphknotenmetastasen über 51 Studien mit insgesamt 11.698 Patienten. Naturgemäß ist die Datentiefe geringer als bei kleineren Fallserien. Jedoch konnte zwischen Primarien der Gl. parotis und der Gl. submandibularis differenziert werden. Erwartungsgemäß war Level II bei Parotiskarzinomen am häufigsten betroffen (62,5 %), gefolgt von Level III (28,6 %), Level I (14,8 %) und Level IV und V (11,4 bzw. 11,7 %). Bei Karzinomen der Submandibulardrüse trat eine okkulte LNM gehäuft in Level I (70,9 %), gefolgt von Level II (58,5 %) und Level III (16,8 %) auf. Deutlicher geringer war eine Beteiligung von Level IV (12,1 %) und Level V (6,8 %; Abb. 3b; [64]). In einer anderen Arbeit konnte diese Beobachtung nicht bestätigt werden. Die Lokalisation des Primarius in der Gl. parotis gegenüber anderen Speicheldrüsen hatte keine prädiktive Aussagekraft für das Auftreten okkulter LNM. Hier war insbesondere die Entität relevant, wobei ein erhöhtes Risiko nur bei Speicheldrüsengangskarzinomen (35,8 % mit okkulter LNM) festgestellt wurde. Allerdings wiesen auch die anderen untersuchten Entitäten erhöhte Raten auf (Adenokarzinom mit 26,8 %, ACC mit 21,1 %, Karzinom ex pleomorphes Adenom mit 22,1 % und Mukoepidermoidkarzinom mit 20,5 %) [65]. Ein Vergleich mit weiteren Analysen der NCDB zeigt ähnliche Raten (Tab. 2; [66, 67]). Hier wird insbesondere der Einfluss des Gradings evident. Low-Grade-Karzinome weisen teilweise eine deutlich geringere Rate okkulter LNM auf [66].

**Tab. 2:** Raten okkulter Lymphknotenmetastasen in Abhängigkeit von der Entität (Raten in Prozent).

Referenz	Vedula S et al. [67]	Tranchito E et al. [65]	Voora R et al. [66]
*Datenbank*	NCDB	NCDB	NCDB
*Kohortengröße*	9405	8689	6977
*Lokalisation*	Gl. parotis	Alle Speicheldrüsen	Gl. parotis
**Mukoepidermoidkarzinom**	13,6	20,5	–
*Low-Grade-Form*	–		6,3
*High-Grade-Form*			22,6
**Azinuszellkarzinom**	7,4	14,9	–
*Low-Grade-Form*	–		3,9
*High-Grade-Form*			22,9
**ACC**	12,2	21,1	–
*Low-Grade-Form*	–		9,6
*High-Grade-Form*			15,7
**Adenokarzinom**	30,0	26,8	–
*Low-Grade-Form*	–		16,2
*High-Grade-Form*			15,7
**Speicheldrüsengangskarzinom**	34,5	35,8	–
*Low-Grade-Form*	–		20,0
*High-Grade-Form*			44,2
**Karzinom ex pleomorphes Adenom**	20,9	22,1	–
*Low-Grade-Form*	–		8,7
*High-Grade-Form*			18,8

*NCDB* National Cancer Data Bank, *ACC* adenoidzystisches Karzinom

Eine spezifische Untersuchung der Rolle von Level V bei einer END für Parotiskarzinome veröffentlichten Katz et al. Hierbei wurden nur originäre Speicheldrüsentumoren eingeschlossen. Eine LNM in Level V lag in 16 % vor und trat nur bei Fällen auf, welche weitere positive Lymphknoten in anterioren Leveln hatten. Die Autoren empfehlen daher eine regelhafte END nur der Level II–IV, falls keine weiteren suspekten Lymphknoten nachweisbar sind (Abb. 3c; [68]).

Eine Analyse 3778 kleiner T1/T2-Karzinome der großen Kopfspeicheldrüsen identifizierte die END als unabhängigen prognostischen Faktor für das gesamt- und das krankheitsspezifische Überleben. Dies traf insbesondere für schlecht differenzierte bzw. undifferenzierte Karzinome sowie Plattenepithelkarzinome zu [69]. Bei intraparotidealen Metastasen kutaner Plattenepithelkarzinome, aber cN0-Status des übrigen Halses, konnte eine okkulte LNM in 30,4 % der Fälle nachgewiesen werden. Ein Vergleich verschiedener Therapiestrategien (END vs. Beobachtung vs. elektive Halsbestrahlung, „elective neck irradiation“, ENI) konnte einen signifikanten Vorteil hinsichtlich der regionalen Kontrolle als auch des krankheitsspezifischen Überlebens für END und ENI gleichermaßen zeigen. Dies war jedoch nur für Fälle mit 1–2 intraparotidealen Metastasen der Fall. Bei ≥ 3 Metastasen war die END einer reinen Radiatio überlegen [70]. Hiervon lässt sich die Möglichkeit eines Verzichts auf eine END bei kleinen und einzelnen intraparotidealen Metastasen ableiten, was v. a. für ältere und multimorbide Patienten von Vorteil sein könnte.

### Sinunasale Karzinome

Die Inzidenz okkulter LNM bei sinunasalen Karzinomen wird mit bis zu 16 % angegeben und ist stark vom histologischen Subtyp abhängig [71, 72]. Für Plattenepithelkarzinome berichtet die jüngste systematische Übersichtsarbeit über eine Rate von 12,5 % für histologisch nachgewiesene okkulte Lymphknotenmetastasen, wobei es sich bei fast der Hälfte davon um eine pN2-Erkrankung handelte [71]. Andere Autoren berichten über Raten von 4,8–7,3 % isolierter Lymphknotenrezidive bei Patienten mit initialem cN0-Sinus-maxillaris-Karzinom [73–75]. Beim adenoidzystischen Sinuskarzinom wurde eine noch höhere Rate von 16 % (4/24) okkulter Lymphknotenmetastasen nach END festgestellt [76]. Bei anderen histologischen Subtypen ist die Inzidenz i. Allg. deutlich niedriger [77–79]. Diese Ergebnisse decken sich mit Daten einer multizentrischen Studie aus der eigenen Arbeitsgruppe. Bemerkenswert ist, dass trotz eines Anteils von 45 % an fortgeschrittenen (T3/T4-)Tumoren 83 % der Patienten keine zervikalen Lymphknotenmetastasen aufwiesen. Die Gesamtrate der okkulten Lymphknotenmetastasen lag bei 6,2 %, wobei diese Metastasen ausschließlich von Plattenepithelkarzinomen (8,4 %) und neuroendokrinen Karzinomen (10 %) stammten.

Zwar sind sich die meisten Autoren einig darüber, dass eine END die lokoregionäre Kontrolle verbessert, jedoch bleibt ein positiver Effekt auf das Gesamtüberleben fraglich [80]. Daher kommt die überwiegende Zahl der Arbeiten zu diesem Thema zum Schluss, dass eine END bei sinunasalen cN0-Karzinomen nicht gerechtfertigt ist, was sich auch in der klinischen Praxis und Entscheidungen interdisziplinärer Tumorboards wiederfindet [74, 81].

Das Ausmaß der therapeutischen Neck-Dissection sollte sich primär nach der klinischen Manifestation der LNM richten. Bei einer auf einzelne Lymphknoten begrenzten Metastasierung sollte die ND zumindest die Level umfassen, welche auch für eine END empfohlen werden. Hierzu konnten in einer größeren retrospektiven Analyse von 299 sinunasalen Malignomen gesicherte LNM am häufigsten in Level II (69 % der N +), gefolgt von Level I (45 % der N +) gefunden werden. Eine Beteiligung retropharyngealer Knoten wurde nur in einer Minderheit der Fälle (17 %) beobachtet und war eher mit großen Tumoren verbunden, bei denen das Epizentrum nicht zuverlässig identifiziert werden konnte [82]. Neuere Arbeiten unterstützen diese Beobachtung, wobei eine LNM in einzelnen Fällen auch in Level III auftreten kann [83]. Bei eindeutigem Verdacht auf Vorliegen einer retropharyngealen Metastasierung ist meist ein strahlentherapeutisches Vorgehen zu favorisieren, da eine gezielte Resektion dieser Lymphknoten kaum möglich ist und ein radikales chirurgisches Vorgehen eine erhebliche Morbidität aufweist [84].

## Alternative Verfahren zur elektiven Neck-Dissection

Insbesondere vor dem Hintergrund der Immuntherapie zeigt sich, dass die prophylaktische Entfernung regionaler Lymphknoten die Prognose nicht zwingend verbessert. Relevant sind v. a. vor Ort verbleibende Gedächtniszellen, die eine zentrale Rolle für die antitumorale Immunität spielen. Eine Lymphknotenentfernung würde somit zugleich einen Großteil der tumorspezifischen Lymphozyten und des ortsständigen Immunsystems entfernen. In einer umfassenden Analyse an Lymphknoten von Kopf-Hals-Tumorpatienten wurde gezeigt, dass sich die Lymphozytensubpopulationen in metastatischen und nichtmetastatischen Lymphknoten deutlich voneinander unterscheiden. So kommen terminal erschöpfte CD8+-T-Zellen v. a. im Primarius und in metastatischen Lymphknoten vor, während in den nichtmetastatischen Lymphknoten noch potenziell differenzierbare T‑Zellen vorhanden sind. Gleichzeitig sind viele dieser Lymphozyten klonal identisch zu den T‑Zellen im Tumormikromilieu. Darüber hinaus konnten die Autoren zeigen, dass eine Immuncheckpointblockade (ICB) zu einer Proliferation und Differenzierung dieser T‑Zellen führt, was in metastatischen Lymphknoten nicht der Fall war. Zusätzlich wurde die Immunreaktion bei Patienten mit metastatischen Lymphknoten gebremst [85]. Dies zeigt wie wichtig es ist, befallene Lymphknoten zu entfernen und gleichzeitig gesunde Lymphknoten zu erhalten. Gleichzeitig wurde gezeigt, dass durch eine ND bzw. Halsbestrahlung im Mausmodell das Ansprechen des Tumors auf eine Immuncheckpointblockade zunichte gemacht wird und sogar das Gesamtüberleben negativ beeinflusst wird [86]. Der Mehrwert einer prophylaktischen ND muss vor diesem Hintergrund und insbesondere in Anbetracht der nun zugelassenen neoadjuvanten Immuntherapie fortgeschrittener Kopf-Hals-Tumoren infrage gestellt werden. Erstrebenswert wäre es also, Mikrometastasen eindeutig zu identifizieren, um präzise die betroffenen Lymphknoten zu behandeln [87].

Ein mögliches Verfahren bietet die sog. Wächterlymphknotenbiopsie („sentinel lymph node biopsy“, SLNB), bei der die ersten drainierenden Lymphknotenstationen des Tumors markiert und gezielt entfernt werden. Das Verfahren wurde in den letzten Jahren insbesondere für Tumoren der Mundhöhle untersucht, und es gibt mittlerweile zahlreiche prospektive Studien, die einen Mehrwert des Verfahrens belegen (Auswahl in Tab. S2). Neben der präziseren Resektion von Lymphknoten mit dem höchsten Risiko für okkulte Metastasen scheint die SLNB auch einen positiven Effekt auf die Lebensqualität zu haben. Hierbei spielt v. a. die Schulterbeweglichkeit eine Rolle, aber auch das optische Ergebnis sowie die Schluckfunktion. Ein systematisches Review zu diesem Thema attestierte den betrachteten Studien jedoch keine überzeugende Evidenz für die Überlegenheit der SLNB [88]. Eine große Analyse der SEER-Datenbank mit 17.019 untersuchten Patienten geht noch einen Schritt weiter und empfiehlt eine Behandlung der Halslymphknoten nur für T1/T2-Zungenkarzinome, nicht aber für andere Lokalisationen, da hier kein Unterschied hinsichtlich des Überlebens zwischen der Gruppe mit und der Gruppe ohne END bestand. Allerdings wurde hier nicht weiter nach dem Ausmaß der ND differenziert [89]. Die Rolle der SLNB wurde auch für andere Lokalisationen untersucht. Eine Metaanalyse konnte auch für Larynx- und Hypopharynxkarzinome eine sehr gute Sensitivität von 0,94 mit einem negativen prädiktiven Wert von 0,97 berechnen. Die Problematik bei Karzinomen in diesen Lokalisationen liegt v. a. in der praktischen Durchführung der Tracerinjektion [90]. Gut geeignet für eine SLNB scheinen sinunasale Karzinome zu sein. Für ein alternatives Verfahren zur END spricht die bekannt niedrige Rate okkulter Lymphknotenmetastasen. Bei T1/T2-Plattenepithelkarzinomen des Sinus maxillaris wurde bei 5 von 47 Patienten eine okkulte Metastase in einem SLN gefunden, was in der entsprechenden retrospektiven Studie sogar zu einer besseren Sensitivität als der verglichenen END führte (83,3 vs. 64,3 %) [91]. Eine Interimsanalyse einer prospektiven Studie der eigenen Arbeitsgruppe konnte eine Detektionsrate von 18 % für sinunasale Plattenepithelkarzinome v. a. der Nasenhaupthöhle demonstrieren [92].

Neben der chirurgischen und histologischen Expertise spielen v. a. die Visualisierung und intraoperative Detektion eine zentrale Rolle. Grundsätzlich gibt es 2 Verfahren zur Markierung des SLN: die Injektion eines Farbstoffs oder eines Radiokolloids, meist Technetium-99 m (^99^mTc). Die Verfahren können darüber hinaus kombiniert werden. In den letzten Jahren wurden vermehrt Techniken mit Fluoreszenzfarbstoffen getestet. Eine Studie im Tiermodell konnte eine 98 %ige Übereinstimmung zwischen dem Fluoreszenzlabel IRDye800CW und dem Radiolabel Gallium-68 (^68^Ga) nachweisen [93]. In 2 Pilotstudien an Patienten mit Mundhöhlenkarzinomen zeigten sich vielversprechende Detektionsraten von 1,2–3,1 SLN pro Patient bei Verwendung einer reinen Fluoreszenzmarkierung mit Indozyaningrün (ICG). Bemerkenswert ist, dass die SLN bereits innerhalb von 3–5 min nach der Injektion detektiert werden konnten und somit eine intraoperative Markierung möglich ist [94, 95]. Eine weitere Möglichkeit bietet die i.v.-Verabreichung von Tracern, die sich gezielt an Tumorzellen anlagern, z. B. ^99^mTc-PSMA (prostataspezifisches Membranantigen) bei Prostatakarzinommetastasen [96]. Eine Studie mit einem Konjugat aus Panitumumab und IRDye800CW zeigte, dass dieses Verfahren der klassischen Radiokolloidmarkierung sogar überlegen sein kann. Die Fluoreszenzintensität in LNM war um bis zu 800 % höher als in nichtbetroffenen Lymphknoten. Im direkten Vergleich mit einer ^99^mTc-basierten Markierung konnte Panitumumab-IRDye800CW zwei metastatische Knoten identifizieren, die mittels Gammakamera nicht erkannt wurden [97].

Nach einer SLNB ist eine verlässliche histopathologische Aufarbeitung des Resektats von zentraler Relevanz. Hier gibt es nun 2 Wege. Zum einen besteht die Möglichkeit der Schnellschnittdiagnostik (auch Gefrierschnitt), um noch intraoperativ zu entscheiden, ob eine Erweiterung auf eine ND indiziert ist, zum anderen kann die Schnittstufendiagnostik abgewartet werden und dann ggf. zweizeitig eine ND durchgeführt werden. Eine größere Metaanalyse hat die diagnostische Güte der Gefrierschnittmethode untersucht und eine eher mäßige Sensitivität von 0,71 errechnet. Dementsprechend lag die Falsch-negativ-Rate bei 34,2 %. Die Autoren empfehlen daher klar eine zusätzliche Stufendiagnostik zur sicheren Detektion okkulter LNM [98].

Noch weniger invasiv als eine SLNB ist die indirekte Detektion maligner Zellen durch eine Blutuntersuchung („liquid biopsy“, LB). Auch diese Methode wurde im Zusammenhang mit der Detektion okkulter LNM untersucht und als möglicher Ersatz für eine END geprüft. Bei der LB werden u. a. zirkulierende Tumorzellen (CTC) aus dem Blut gefiltert. Das Problem hierbei ist, dass diese Zellen nur sehr vereinzelt vorkommen und ihre Isolierung daher erschwert ist. Ein häufiger vorkommender Zelltyp ist die sog. zirkulierende Hybridzelle (CHC). Hierbei handelt es sich um eine Fusion aus neoplastischer Zelle und Makrophage mit spezifischer Oberflächenmarkerexpression. Die Zellen sind gut zu identifizieren und kommen deutlich häufiger als klassische CTC vor. Höhere CHC-Level waren in einer Studie an 22 Patienten mit Mundhöhlenkarzinomen signifikant mit dem Vorliegen okkulter Lymphknotenmetastasen assoziiert. Bei einem Cut-off-Wert von ≥ 20 CHC/50.000 Zellen ergab sich eine Sensitivität von 95 % mit einer Falsch-negativ-Rate von 0 % [99]. Noch einfacher erscheint die Analyse des Differenzialblutbilds mit Bildung spezifischer Quotienten, wobei der bekannteste das Neutrophilen-Lymphozyten-Verhältnis (NLR) ist. Allerdings sind die Daten hier sehr heterogen, reichen von einem Cut-off-Wert bis zu einem Wertekorridor, und so erscheint die Methode als wenig verlässlich [100–104].

Eine weitere Alternative zur END ist die Ableitung einer möglichen LNM aus der Gewebeuntersuchung des Primarius anhand von Biomarkern. Die Analyse der mRNA-Expression von „glycerol-3-phosphate dehydrogenase 1‑like“ (GPD1L) und von „hypoxia-inducible factor-1α“ (HIF1α) in T1/T2-Kopf-Hals-Tumoren zeigte, dass eine niedrige GPD1L- und eine hohe HIF1α-Expression mit einer signifikant höheren verzögerten Metastasierungsrate assoziiert ist. Die GPD1L- und HIF1α-Proteinexpression konnte Lymphknotenmetastasen zudem präziser vorhersagen als das WINTER-Hypoxie-Genpanel (Falsch-negativ-Rate bei der Vorhersage von Metastasen: 8,1 % gegenüber 26,4 %) [105]. Andere Autoren postulieren weitere Gensignaturen im Primärtumor zur Vorhersage einer LNM. Die jeweilige Sensitivität nach Optimierung der Signatur erreichte hier jeweils 100 %, die Spezifität reichte von 81–91,7 % [106, 107]. Trotz dieser teilweise sehr überzeugenden diagnostischen Qualität hat sich bislang keine der Signaturen im klinischen Alltag durchgesetzt. Hier wäre zunächst eine Vergleichsanalyse der verschiedenen Signaturen notwendig, welche diejenige mit der besten Performance identifiziert, um dann eine große Phase-III-Studie auf den Weg zu bringen.

## Detektion metastatischer Lymphknoten

Auf dem Weg zu mehr Präzision bei der Behandlung der Halslymphknoten ist ein exaktes Staging unerlässlich. Moderne Techniken und Entwicklungen in der Bildgebung, aber auch der Einsatz von künstlicher Intelligenz sind hierbei zu betrachten. Schlussendlich kann eine gezielte Therapie und v. a. auch eine weniger radikale Therapie nur sicher durchgeführt werden, wenn der Chirurg vor dem Eingriff Zugang zu bestmöglicher Datenqualität hat. Dies ist unerlässlich im präoperativen Setting, kann aber auch intraoperativ wertvolle Hinweise auf die Notwendigkeit einer Erweiterung der ND liefern.

### Präoperativ

Die Entscheidung, ob eine therapeutische oder eine elektive ND durchgeführt werden muss, hängt maßgeblich von der Güte des prätherapeutischen Stagings ab. Zudem kann durch eine exakte Einschätzung der Größe des Primarius u. U. eine END vermieden werden. Es werden hier zunehmend verbesserte Bilderkennungsalgorithmen für verschiedene Modalitäten entwickelt.

Bei Mundhöhlenkarzinomen mit einer geringen DOI kann auf eine END verzichtet werden. Diese Information kann mittels hochfrequenten intraoralen Ultraschalls bereits in der Planungsphase erhoben werden. Eine entsprechende Studie an 41 Patienten mit Zungenkarzinomen ergab eine Sensitivität von 92,31 % mit einer Spezifität von 82,14 % für die Vorhersage einer DOI ≥ 4 mm. Zudem lag der mittlere systematische Fehler unter dem Wert, der für eine Abschätzung der DOI mittels Magnetresonanztomographie (MRT) auftrat [108].

Die Rolle der Positronenemissionstomographie-Computertomographie (PET-CT) bei der Detektion okkulter Lymphknotenmetastasen wurde in einem systematischen Review von 2024 aufgearbeitet. In der gepoolten Analyse aller Studien konnte eine Sensitivität von 0,71 und eine Spezifität von 0,9 berechnet werden. Die Detektionsrate okkulter LNM ist somit zu niedrig, um sich darauf bei einer Entscheidung für oder gegen eine END zu verlassen [109]. Auch im Direktvergleich mit Ultraschall, Computertomographie (CT) und MRT scheint die PET-CT nicht überlegen zu sein. Die beste diagnostische Genauigkeit hatte hier die Sonographie mit 86,6 % [110].

Eine spezielle Herausforderung stellt die Beurteilung einer residuellen LNM nach primärer Radiochemotherapie dar. Hier wurde kürzlich nachgewiesen, dass diese insbesondere durch die Kombination aus CT und Sonographie mit einer Genauigkeit von 82,7 % vorhergesagt werden kann [111].

Die Bilddatenanalyse wird zunehmend in verschiedene Modelle künstlicher Intelligenz (KI) integriert. Darüber hinaus können Modelle auch ausschließlich mit klinischen Daten trainiert werden, um das Auftreten einer LNM vorherzusagen (Tab. S3). Im Bereich der Mundhöhle wird die DOI häufig als Vergleichswert herangezogen, um die diagnostische Güte eines Modells zu bewerten. In einem Vergleich verschiedener Machine-Learning(ML)-Algorithmen erwies sich die vergleichsweise einfache Lasso-logistische Regression als überlegen und erreichte eine Fläche unter der Kurve („area under the curve“, AUC) von 0,782 im Vergleich zu 0,564 und 0,611 für einen DOI-Cut-off-Wert von 3 bzw. 4 mm [112]. Eine vergleichbare Studie, die zusätzliche klinische Eingangsparameter einbezog, identifizierte den XGBoost-Algorithmus als überlegen gegenüber anderen Modellen (inkl. DOI). Die erzielte AUC betrug 0,84 [113]. Insbesondere bei der heterogenen Gruppe der Speicheldrüsenkarzinome wäre eine KI-gestützte Entscheidungshilfe wünschenswert. Ein entsprechendes Modell wurde anhand von SEER-Daten entwickelt und trainiert. Die beiden angewandten Algorithmen waren mit einer AUC von 0,7 und 0,71 ähnlich gut. Die Spezifität beider Modelle war mit 0,9 und 0,83 ebenfalls zufriedenstellend, allerdings zeigte sich eine niedrige Sensitivität von 0,27 und 0,38. Somit gibt es hier noch weiteren Verbesserungsbedarf. Eine Möglichkeit hierzu liegt in der Einbindung von Bilddaten in die Modelle. Aktuell beschränken sich die Arbeiten meist auf eine Bildgebungsmodalität. In einer Studie an dimensionsreduzierten MRT-Bildern wurde in 74,1 % der Fälle der Nodalstatus korrekt detektiert, was zu einer AUC von 0,802 führte [114]. Eine neuere Arbeit analysierte die vollständigen MRT-DICOM-Datensätze (Digital Imaging and Communications in Medicine, DICOM) von 723 Patienten mit einem dreistufigen Deep-Learning-Modell. Die Autoren konnten eine AUC von 0,99 in der primären Kohorte und von immerhin 0,81 in der externen Validierungskohorte erzielen [115]. Noch bessere Werte wurden mit Modellen erzielt, die CT-Daten nutzen (AUC 0,824–0,98) [116, 117]. Die Modelle sind jedoch noch nicht ausgereift genug, und es bedarf weiterer Studien, um das optimale KI-Modell und die optimalen Eingangsdaten zu definieren.

### Intraoperativ

Um das Ausmaß der ND intraoperativ dynamisch anzupassen, wäre eine verlässliche Detektion einer LNM im Situs notwendig. Die konventionelle Methode hierzu ist die „staged ND“, die in einer intraoperativen Schnellschnittuntersuchung der entfernten Lymphknoten besteht. Abhängig vom Ergebnis wird die ND von einer SND auf eine MRND ausgedehnt [118]. Dies ist zum einen zeitintensiv und zum anderen fehleranfällig, wenn man bedenkt, wie wenig verlässlich die Schnellschnittdiagnostik hinsichtlich der Detektion von Mikrometastasen ist [98].

Eine Möglichkeit, metastatische Lymphknoten intraoperativ zu detektieren, bietet die ultraschnelle konfokale Fluoreszenzmikroskopie (UFCM). Hierbei werden resezierte Lymphknoten mit einem Fluoreszenzfarbstoff inkubiert und von Pathologen begutachtet. In einer Pilotstudie mit 44 Patienten wurde eine sehr gute Konkordanz zwischen 2 verblindeten Pathologen von 95,5 % erreicht. Gegenüber dem Goldstandard (fixierte und in Paraffin eingebettete Resektate, gefärbt mit Hämatoxylin/Eosin/Safran und Immunhistochemie für Zytokeratin) zeigte sich die UFCM mit einer Spezifität von 95,5 %. Allerdings lag die Sensitivität für das Verfahren nur bei 76,7 %. Die Autoren schlussfolgern, dass dies durch Implementierung weitere technischer Verfahren (z. B. KI) verbessert werden kann [119]. Durch die intraoperative Gabe von Indozyaningrün können metastatische Lymphknoten bereits in situ detektiert werden [120]. Die Identifikation von Tumorgewebe kann besonders in der Rezidivsituation durch die Vorbehandlung erschwert sein. In dieser Konstellation konnte gezeigt werden, dass hinsichtlich der Intensität der Fluoreszenz kein relevanter Unterschied nach Radiotherapie besteht. Allerdings ist der Anteil von Tumorgewebe innerhalb des Lymphknotens ausschlaggebend für die Intensität der Fluoreszenz und die Technik daher für die Detektion von Mikrometastasen eher ungeeignet [121].

## Risikoabschätzung durch Neck-Dissection

Ein zentrales Argument für die Durchführung einer END ist die dadurch mögliche Risikoabschätzung, welche erst durch ein histopathologisches Staging präzise wird. Dafür müssen mehrere Punkte beachtet werden. Vor allem ist die Ausbeute der ND, sprich die Anzahl beurteilbarer Lymphknoten, relevant. Die minimale Anzahl für eine aussagekräftige ND (Lymphknotenausbeute, „lymph node yield“, LNY) wurde für Mundhöhlenkarzinome mit 18 Knoten pro Halsseite bestimmt [122]. Diese Anzahl wird seither von den meisten Autoren als Benchmark auch für Tumoren anderer Lokalisationen im Kopf-Hals-Bereich angesehen. Mögliche Einflussfaktoren für eine entsprechende Ausbeute wurden von Hintze et al. untersucht. Jedoch hatte weder das Ausmaß der ND (3 vs. 4 vs. 5 Level) noch das Patientenalter, die Lokalisation des Primarius, der HPV-Status oder der Ausbildungsstatus des Pathologen einen signifikanten Einfluss auf die Anzahl resezierter Lymphknoten. Den einzigen signifikanten Faktor stellte eine präoperative Radiotherapie dar. Hierdurch war die Ausbeute im Mittel um 14,78 Lymphknoten geringer [123]. Diese Beobachtung wird von weiteren Arbeiten gestützt. Ein positiver Effekt auf die LNY wurde für ein höheres Körpergewicht sowie einen höheren Body-Mass-Index (BMI), ein Patientenalter > 40 und männliches Geschlecht gemessen [124, 125]. Darüber hinaus können große Metastasen mit extrakapsulärem Wachstum (ENE+) die LNY pro Level reduzieren [126]. Ein klares Protokoll zur Aufarbeitung und Begutachtung von ND-Präparaten kann die LNY wiederum um etwa 30 % erhöhen [127]. Ein entsprechendes Datenset wurde von Bullock et al. zusammengestellt (Tab. 3; [128]).

**Tab. 3:** Vorschlag für Inhalte und Dokumentation einer strukturierten histopathologischen Befundmitteilung (nach [128]).

Kernelement (gefordert)	Nebenelemente (empfohlen)
Eingesandtes Gewebe	Operationsverfahren
Histologische Tumorentität	Primärtumorlokalisation
Anzahl der untersuchten Lymphknoten (pro Level)	R‑Status
Anzahl positiver Lymphknoten (pro Level)	Histologische oder molekularpathologische Zusatzuntersuchungen
Maximale Ausdehnung des größten befallenen Lymphknotens	Durch ENE infiltrierte, nichtlymphatische Strukturen
Maximale Ausdehnung der größten Weichgewebsmetastase (für jede Seite des Halses, falls zutreffend)	Größte Ausdehnung der ENE in mm
ENE (vorhanden/nicht identifiziert; mikro- oder makroskopisch)	Anzahl ENE+-Lymphknotenmetastasen
Weichgewebsmetastasen (vorhanden/nicht identifiziert)	
N‑Kategorisierung der regionalen Lymphknoten	

*ENE* extranodale Ausbreitung („extra nodal extension“)

Die Frage nach der optimalen Anzahl dissezierter Lymphknoten ist jedoch weiter Gegenstand wissenschaftlicher Untersuchungen. Die Vergleichbarkeit der Arbeiten unterliegt dabei einer starken Verzerrung durch uneinheitliche chirurgische Vorgehensweisen, Unterschiede in der Qualität des präoperativen Stagings und einen wenig standardisierten pathologischen Befundbericht [125]. Neuere Studien zeigen eine verlässliche Prognoseabschätzung schon bei geringeren LNY. Für supraglottische T1/T2-Larynxkarzinome wird eine LNY von > 10 als ausreichend erachtet, sofern ein pN0-Stadium vorliegt [129]. Eine Analyse von 579 Mundhöhlenkarzinomen errechnete mittels eines komplexen, mehrstufigen Testkonzepts eine LNY von > 15 als optimal für die Abschätzung von krankheitsfreiem Überleben, lokoregionären Rezidiven und isolierten Lymphknotenrezidiven [130]. Auch für cN0-Oro- und Hypopharynxkarzinome scheint eine minimale LNY von 15 Knoten für die prognostische Abschätzung des krankheitsspezifischen Überlebens und der regionalen Kontrolle ausreichend zu sein. Allerdings konnte dies nicht für Larynxkarzinome bestätigt werden [131]. Bei Tumoren in dieser Lokalisation wurde eine gemeinsame minimale LNY für beide Halsseiten ermittelt. Hier erwies sich ein Cut-off-Wert von 24 als prognostisch aussagekräftig für das Gesamtüberleben und 26 für das krankheitsfreie Überleben [56].

Ein weiterer Parameter, der sich als prognostisch etabliert hat, ist die „lymph node ratio“ (LNR). Hierbei handelt es sich um die Anzahl positiver Lymphknoten geteilt durch die Gesamtzahl dissezierter Lymphknoten [132]. In einem Direktvergleich mit der LNY für Mundhöhlenkarzinome war die LNR prognostisch überlegen. Hier wurde ein signifikant verbessertes Überleben für Patienten mit einer LNR ≤ 6,6 % berechnet [133]. In einer Arbeit zur LNR bei p16+-Oropharynxkarzinomen wurde ein signifikant schlechteres krankheitsfreies Überleben erst ab Werten ≥ 12,9 % beobachtet, was höchstwahrscheinlich die insgesamt bessere Prognose auch bei Vorliegen einer LNM in dieser Entität widerspiegelt [134]. In einem Vergleich von insgesamt 5 verschiedenen Modellen zur Prognoseeinschätzung anhand der Lymphknoten am Beispiel von Hypopharynxkarzinomen war die LNR die exakteste Methode. Zum Vergleich wurden LNY, pN-Status, Anzahl positiver Lymphknoten und der Logarithmus der Odds Ratio positiver Lymphknoten herangezogen. In der Arbeit wurde eine Risikoklassifizierung anhand der LNR von R0–R3 vorgeschlagen und in ein prognostisches Nomogramm integriert [135]. Ausgehend von der LNR wurden noch weitere Verfeinerungen („lymph node level ratio“, „weighted lymph node ratio“ usw.) getestet, wobei die jeweiligen Autoren das vorgeschlagene Modell meist als überlegen gegenüber den bisherigen Modellen ansehen [136–139].

Für andere maligne Tumoren ist der Logarithmus der Odds Ratio (LODDS) eine etablierte Berechnung zur Prognoseabschätzung. Dieser berechnet sich für das aus einer LNM resultierende Risiko als der natürliche Logarithmus der LNR. Mehrere Arbeiten zu diesem Thema konnten den LODDS als unabhängigen prognostischen Wert definieren. Jedoch gibt es hier deutliche Unterschiede je nach Lokalisation der Tumoren. So scheint der LODDS bei Mundhöhlenkarzinomen besonders relevant für pN0 und eine inadäquat durchgeführte ND zu sein [140]. Dies konnte an einer weiteren monozentrischen Kohorte allerdings nicht bestätigt werden [141]. In einer Analyse verschiedener Tumorlokalisationen und Daten aus verschiedenen Kliniken war der LODDS deutlich besser für eine Prognoseabschätzung des Gesamtüberlebens geeignet als die LNR oder die Anzahl positiver Lymphknoten [142].

Insgesamt herrscht auch bei den weiterführenden Berechnungen ausgehend von der Anzahl der dissezierten Lymphknoten keine Einigkeit hinsichtlich der optimalen Cut-off-Werte und teilweise sogar hinsichtlich des Stellenwerts als prognostischer Parameter.

Eine weitere prognostische Information, die i. d. R. nur durch eine ND zu erlangen ist, ist das Vorliegen eines extrakapsulären Wachstums der Lymphknotenmetastasen (ENE, „extranodal extension“). Neben dem Resektionsstatus des Primarius ist diese Information bisher entscheidend, ob eine konkomitante Chemotherapie im Rahmend er adjuvanten Bestrahlung eingesetzt wird. Allerdings wird die Relevanz sowohl von ENE als auch einer R1-Resektion durch Ergebnisse der Studien RTOG 9501 und EORTC 22931 infrage gestellt [143].

## Fazit für die Praxis

- Die Neck-Dissection (ND) stellt nach wie vor einen essenziellen Bestandteil der chirurgischen Therapie von Kopf-Hals-Malignomen dar.
- Gemäß der aktuellen Studienlage besteht die Möglichkeit, das Ausmaß sowohl der therapeutischen als auch der elektiven ND teilweise zu reduzieren und zu präzisieren. Dieser Ansatz erweist sich insbesondere vor dem Hintergrund der Immuntherapie als vorteilhaft.
- Die Sentinellymphknotenbiopsie stellt eine alternative Option zur elektiven ND dar. Derzeit liegt gesicherte Evidenz allerdings nur für frühe Mundhöhlenkarzinome vor.
- Die Voraussetzung für eine zunehmende Präzisierung und Reduzierung des Umfangs der ND ist eine präzise prä- bzw. intraoperative Diagnostik. Es besteht weiterer Forschungsbedarf, um einerseits bildgebende Verfahren und andererseits den Einsatz künstlicher Intelligenz zu validieren.
- In Bezug auf die Prognoseabschätzung und die Planung einer eventuellen adjuvanten Therapie ist der Erkenntnisgewinn aus einer ND der Goldstandard. Eine zunehmende Präzisierung wird u. a. durch den Einsatz mathematischer Formeln ermöglicht, hat aber bislang noch keine klinische Relevanz.

## Supplementary Information

Tabelle S1: Kontralaterale okkulte lymphonodale Metastasierung (LNM) bei Oropharynxkarzinomen; HPV humanes Papillomavirus. Tabelle S2: Auswahl prospektiver Studien zur Sentinel-Lymph-Node-Biopsie (SLNB) bei Mundhöhlenkarzinomen; OSCC „oral squamous cell carcinoma“; END elektive Neck-Dissection. Tabelle S3: Künstliche-Intelligenz(KI)-Modelle zur Vorhersage des Auftretens zervikaler Lymphknotenmetastasen.
