# Supplementary material for: Neck dissection—more, less, targeted?
Source: HNO. 2025 Dec 18;74(7):445–61. [Article in German] doi: 10.1007/s00106-025-01696-z (PMC13303559; doi:10.1007/s00106-025-01696-z)
Supplement: Supplementary file 1 — Tabelle S1: Kontralaterale okkulte lymphonodale Metastasierung (LNM) bei Oropharynxkarzinomen; HPV humanes Papillomavirus. Tabelle S2: Auswahl prospektiver Studien zur Sentinel-Lymph-Node-Biopsie (SLNB) bei Mundhöhlenkarzinomen; OSCC „oral squamous cell carcinoma“; END elektive Neck-Dissection. Tabelle S3: Künstliche-Intelligenz(KI)-Modelle zur Vorhersage des Auftretens zervikaler Lymphknotenmetastasen. [file 106_2025_1696_MOESM1_ESM.pdf]

supplementary material

Referenz	Jahr	Gesamtrate okkulter kontralateraler Metastasen	Rate bei ipsilateral cN0	Rate bei ipsilateral cN+	Einfluss der Primariuslokalisation	Einfluss von HPV	Einfluss des T-Stadiums
Knopf A et al. [42]	2020	0,6 %	n/a	n/a	-	n/a	-
Smith A et al. [39]	2021	8 %	0 %	9 %	Nur Mittelliniennähe relevant, Zungengrund nicht	Nur HPV+ Fälle eingeschlossen	n/a
Cho K et al. [38]	2022	11,1 %	n/a	n/a	Nur Tonsillenkarzinome eingeschlossen	Nur HPV+ Fälle eingeschlossen	signifikante Korrelation mit höheren T-Stadien
Zhang Y et al. [3]	2022	n/a	0,68 %	11,49 %	5.57% bei ipsilateral cN0 und 13.04% bei ipsilateral cN+ Zungengrundkarzinomen	n/a	n/a
Malik A et al. [35]	2023	9,8 %	1,7 %	9,8 %	n/a	n/a	n/a
Kim GJ et al. [36]	2024	8,8 %	0 %	10,7 %	Nur Tonsillenkarzinome eingeschlossen	Nur HPV+ Fälle eingeschlossen	keine Relevanz

Punjabi N et al. [37]	2025	10 %	1 %	12 %	Nur Tonsillen karzinom e eingesch lossen	n/a	8% für cT1/2, 19 % für cT3/4

Tabelle S1. Kontralaterale okkulte LNM bei Oropharynxkarzinomen. HPV, Humanes Papillomavirus.

Referenz	Jahr	Studiendesign	Primärer Endpunkt	Kernaussage
Fang Q et al.*	2024	open-label, Einarmig	Rate metastatischer Lymphknoten jenseits des SLN	Hohes Risiko für Nicht-SLN-Metastasen bei DOI > 4 mm
Doll C et al.**	2023	open-label, Einarmig	SLN Detektionsrate	Auch bei Z.n. ND kann eine SLNB das Staging verbessern
Hasegawa et al.***	2021	open-label, randomisiert, Zweiarmig	3-Jahres-Gesamtüberleben	SLNB ist einer END bei T1/T2 OSCC nicht unterlegen und gleichzeitig weniger invasiv
Garrel R et al. +	2020	open-label, randomisiert, Zweiarmig	2-Jahres-Lymphknotenrezidivfreies Überleben	SLNB ist einer END bei T1/T2 OSCC/OPSCC nicht unterlegen bei besserem funktionellem Ergebnis

Tabelle S2. Auswahl prospektiver Studien zur Sentinel Node Biopsie (SLNB) bei Mundhöhlenkarzinomen. OSCC, oral squamous cell carcinoma; END, elective Neck dissection.

Referenz	Jahr	Tumorlokalisation	Methode	Parameter
Yuan Y et al. [114]	2021	Zungenkarzinome	Machine Learning Algorithmen (LR, random for-	Extrahierte und dimensionsreduzierte MRT-Daten

			est, naïve Bayes, SVM, AdaBoost, and neural network	
Farrokhian N et al. [113]	2022	Mundhöhlenkarzinome	Machine Learning Algorithmus (XGBoost)	Alter, Geschlecht, ethnische Zugehörigkeit, Body-Mass-Index, Nikotin, Tumorlokalisation, Tumorgröße, DOI, Muskelinvasion, Dysplasie, Grading, R-Status, PNI, LVI
Taku N et al. [116]	2022	HPV+ Oropharynxkarzinome	Deep learning convolutional neural network	CT DICOM Datensätze
Costantino A et al.[++]	2023	Speicheldrüsenkarzinome	Machine Learning Algorithmen (random forest, XGBoost)	Alter bei der Diagnose, Geschlecht, ethnische Zugehörigkeit, Familienstand, Urbanisierungsgrad, Haushaltseinkommen, Diagnosejahr, Primärtumorlokalisation, Tumorgrad, Tumorhistologie, Tumorgröße (in mm), T-Klassifikation
Han W et al. [117]	2024	Zungenkarzinome	Machine Learning und Deep Learning Algorithmen (SVM, kNN, random forest, extra trees, XGBoost, LightGBM, MLP, LR, ResNet50)	Extrahierte und präprozessierte CT-Bilder
Sun N et al. [112]	2025	Bukkale Mundhöhlenkarzinome	Lasso-logistische Regression	Nikotin, DOI, Differenzierung, Ki67, PNI, R-Status
Yang L et al. [115]	2025	Mundhöhlenkarzinome	3-Stufiges Deep Learning Modell	MRT DICOM Datensätze

Tabelle S3. KI-Modelle zur Vorhersage des Auftretens zervikaler Lymphknotenmetastasen.

Referenzen nicht im Hauptteil angeführt:

* Fang Q, Yuan J, Zhang X, Dai L, Luo R, Huang T. Omitting elective neck dissection in cT1/2N0 oral squamous cell carcinoma with sentinel lymph node metastasis: A prospective study. Oral Oncol. 2025 Feb;161:107149. doi: 10.1016/j.oraloncology.2024.107149.

** Doll C, Bigus S, Hofmann E, Mrosk F, Steffen C, Thiele F, Voss J, Kreutzer K, Amthauer H, Heiland M, Koerdt S. Sentinel lymph node biopsy in recurrent or secondary oral squamous cell carcinoma after previous neck dissection. Oral Oncol. 2023 Oct;145:106494. doi: 10.1016/j.oraloncology.2023.106494.

*** Hasegawa Y, Tsukahara K, Yoshimoto S, Miura K, Yokoyama J, Hirano S, Uemura H, Sugasawa M, Yoshizaki T, Homma A, Chikamatsu K, Suzuki M, Shiotani A, Matsuzuka T, Kohno N, Miyazaki M, Oze I, Matsuo K, Kosuda S, Yatabe Y; HNCMM Research Group. Neck Dissections Based on Sentinel Lymph Node Navigation Versus Elective Neck Dissections in Early Oral Cancers: A Randomized, Multicenter, and Noninferiority Trial. J Clin Oncol. 2021 Jun 20;39(18):2025-2036. doi: 10.1200/JCO.20.03637.

+ Garrel R, Poissonnet G, Moyà Plana A, Fakhry N, Dolivet G, Lallemant B, Sarini J, Vergez S, Guelfucci B, Choussy O, Bastit V, Richard F, Costes V, Landais P, Perriard F, Daures JP, de Verbizier D, Favier V, de Boutray M. Equivalence Randomized Trial to Compare Treatment on the Basis of Sentinel Node Biopsy Versus Neck Node Dissection in Operable T1-T2N0 Oral and Oropharyngeal Cancer. J Clin Oncol. 2020 Dec 1;38(34):4010-4018. doi: 10.1200/JCO.20.01661.

++ Costantino A, Canali L, Festa BM, Kim SH, Spriano G, De Virgilio A. Development of machine learning models to predict lymph node metastases in major salivary gland cancers. Eur J Surg Oncol. 2023 Sep;49(9):106965. doi: 10.1016/j.ejso.2023.06.017.
